# Homebrewed psilocybin: can new routes for pharmaceutical psilocybin production enable recreational use?

**DOI:** 10.1080/21655979.2021.1987090

**Published:** 2021-10-26

**Authors:** William J. Gibbons, Madeline G. McKinney, Philip J. O’Dell, Brooke A. Bollinger, J. Andrew Jones

**Affiliations:** a Department of Chemical, Paper, and Biomedical Engineering, Miami University, Oxford, OH, USA; b Department of Chemistry and Biochemistry, Miami University, Oxford, OH, USA

**Keywords:** Psilocybin, regulation, homebrewed drugs, recombinant DNA technology

## Abstract

Psilocybin, a drug most commonly recognized as a recreational psychedelic, is quickly gaining attention as a promising therapy for an expanding range of neurological conditions, including depression, anxiety, and addiction. This growing interest has led to many recent advancements in psilocybin synthesis strategies, including multiple *in vivo* fermentation-based approaches catalyzed by recombinant microorganisms. In this work, we show that psilocybin can be produced in biologically relevant quantities using a recombinant *E. coli* strain in a homebrew style environment. In less than 2 days, we successfully produced approximately 300 mg/L of psilocybin under simple conditions with easily sourced equipment and supplies. This finding raises the question of how this new technology should be regulated as to not facilitate clandestine biosynthesis efforts, while still enabling advancements in psilocybin synthesis technology for pharmaceutical applications. Here, we present our homebrew results, and suggestions on how to address the regulatory concerns accompanying this new technology.

## 1. Introduction:

Since the 1970s, genetic engineering and biomanufacturing technology utilizing recombinant DNA have led to many advancements in medicine [1,2], agriculture [3,4], and energy [5,6] and continue to be an enabling technology for cutting edge discoveries [7]. Despite the widespread impact of recombinant DNA technologies, public concern and controversy still remain [8–12].

Recently, recombinant DNA has been used to produce psilocybin, the chemical found in psychedelic mushrooms that causes a hallucinogenic response upon ingestion. This method for producing psilocybin became possible after the Hoffmeister group reported the metabolic pathway for the biosynthetic production of psilocybin [13].

Natural sources of psilocybin such as *Psilocybe sp*. and related mushrooms have been historically consumed by some native populations for religious and ceremonial purposes [14–16]. In modern times, psilocybin has been popularized by the recreational use of these so called ‘magic’ mushrooms, with one study reporting nearly 10% of adults in the United States (US) having used psilocybin at some point in their lifetime [17]. Psilocybin has returned to the spotlight due to recent positive results from clinical trials for the treatment of a variety of neurological issues, including treatment-resistant depression, post-traumatic stress disorder (PTSD), cancer related anxiety, substance abuse, and more [18–21].

Due to these promising clinical results, pharmaceutical companies are investigating more cost-effective means to produce psilocybin. Growing and harvesting *Psilocybe* mushrooms shows limited economic feasibility due to the slow production process and high product variability [22]. Current production of the psilocybin active pharmaceutical ingredient for clinical trials is achieved through traditional chemical synthesis, which despite recent advancements [23,24], still requires a complex, multi-step synthesis process that is hindered by low yield and high production costs [25–27]. The biosynthesis of psilocybin, made possible by recombinant DNA technology, has recently emerged as a competing technology for psilocybin production. The Hoffmeister and Valiante groups were the first to report the *in vivo* production of psilocybin from the eukaryotic fungal host, *Aspergillus nidulans* [28]. Subsequently, the Jones group produced psilocybin *in vivo* in the prokaryotic host, *E. coli*, from 4-hydroxyindole [29]. The Borodina group has since demonstrated *de novo* synthesis of psilocybin from glucose in *Saccharomyces cerevisiae* [30]. Each of these methods have advantages and disadvantages and will require further advances to enable large-scale production using current good manufacturing practices (cGMP).

Although the approval and use of psilocybin as a legal pharmaceutical product could potentially bring relief to millions of people that deal with severe anxiety, PTSD, and depression, there are important safety and regulatory concerns associated with the realization of this potential. Psilocybin is currently highly regulated and was classified as a Schedule I controlled substance in 1970 as part of the US Controlled Substances Act after being deemed to have ‘no currently accepted medical use and high potential for abuse’ [31]. However, if the United States Food and Drug Administration (FDA) approves psilocybin for medical use in the future, experts speculate that it would be reclassified as a Schedule IV compound, supported by recent data indicating high safety and low addictive properties [32]. With the potential for less government regulation and new methods enabling biosynthetic psilocybin production, there is increased potential for clandestine production of psilocybin for recreational use. The concern is that an individual could acquire or (re)create a microbial strain containing recombinant DNA that enables rapid and high-level psilocybin production, and the use of this strain to produce psilocybin for recreational use could pose a threat to public health and safety.

The threat of recreational production of regulated compounds using recombinant DNA technology is not specific to psilocybin, or psychedelics as a whole. Previous to psilocybin biosynthesis, metabolic pathways were discovered that enabled production of several opium alkaloids and a range of cannabinoids in *S. cerevisiae* [33,34]. Although impressive, the production levels resulting from these studies were much below the biologically relevant levels, and thus were of less immediate concern [35]. The increasing number of proof-of-principle studies in this research space is motivation for further discussion surrounding the proper regulatory and control mechanisms that enable further scientific discovery, while limiting the potential for misuse and abuse.

Just as popular interest in the cultivation of *Psilocybe cubensis* mushrooms at home increased with the availability of information [36,37], the successful fermentation-based production of psilocybin has prompted the following question: ‘Could someone with basic skills in fermentation produce relevant quantities of psilocybin, using commonly available items, assuming they could acquire access to the specifically engineered microbial catalyst?’ That is the question this work aims to answer. Here, we show the successful production of biologically relevant quantities of psilocybin under mimicked homebrew conditions. Based on this finding, we provide comprehensive recommendations focused on how to limit clandestine biosynthesis efforts while maintaining the ability to perform valuable research into the manufacture and clinical validation of this powerful drug.

## 2. Methods:

*E. coli* BL21 star^TM^ (DE3), containing the psilocybin pathway expression plasmid pPsilo16, was used for all production cultures [29]. A modified version of Andrew’s Magic Media (AMM) was used for all cultivation conditions [38]. Production cultures were induced with 1 mM isopropyl β-D-1-thiogalactopyranoside (IPTG) four hours after inoculation with an overnight culture. The fermentations proceeded for a total of 72 hours.

**Figure 1.**
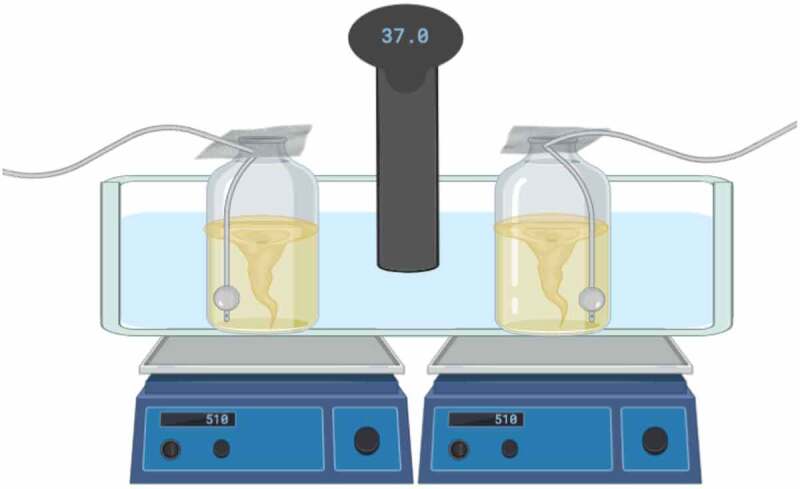
Visual representation of the homebrew experimental setup. Two 500 mL bottles, containing inoculated cultures, magnetic stir bars, and air stones, were placed into a plastic tub filled with water. The air stones were attached to small aquarium air pumps via plastic tubing. The tops of the bottles were covered with aluminum foil. The tub was placed onto two magnetic stir plates, set to 510 RPM. A sous vide, set to 37 °C, was attached to the plastic tub and inserted into the water bath

A rudimentary setup was constructed using low-cost and easily available items (Figure 1). Water bath temperature was controlled at 37 °C using a residential grade sous vide. The parallel cultures were aerated using small aquarium air pumps with attached air stones. The stir rate was set to 510 revolutions per minute (RPM) on a magnetic stir plate. All cultures were grown in 250 mL of AMM in 500 mL glass bottles. Boluses of 4-hydroxyindole (150 mg/L final concentration per bolus) were added initially and as needed to maintain pathway flux toward psilocybin. This resulted in a total of 450 mg/L being added to the Basic Homebrew and Homebrew with Ampicillin Conditions tests and 600 mg/L being added to the Standard Conditions tests. Psilocybin production was quantified via high performance liquid chromatography (HPLC) using methods described previously [29].

Three sets of experimental conditions were tested using six replicates each. First, the cultures were grown using standard sterile technique; all glassware was autoclaved, the media was filter sterilized under flame, all samples were extracted under flame, and ampicillin was used to select for the recombinant strain and limit contamination, referred to here as ‘Standard Conditions’. Next, the glassware was ethanol rinsed, the media was not filter sterilized, samples were extracted without a flame, and ampicillin was not used, referred to here as ‘Basic Homebrew Conditions’. Third, the same setup as Basic Homebrew Conditions was used, except ampicillin was present, referred to here as ‘Homebrew with Ampicillin Conditions’. Statistical analysis was performed using a two-tailed, unpaired t-test and the full dataset and analysis is provided in the supplementary materials.

## 3. Results:

Recently, the biosynthesis of psilocybin has gained much attention due to ongoing clinical studies investigating its therapeutic efficacy for a range of neurological conditions. The success of these biosynthesis studies has made possible the potential for misuse of this recombinant DNA-based technology. Here, we present our evaluation of the potential for an engineered microbial biosynthesis platform to be used in a homebrew style environment to enable the illicit production of the controlled substance, psilocybin. This study presents the motivating evidence to support the feasibility of this scenario and provides a comprehensive analysis of mitigating measures that can be taken to limit the clandestine use of this technology.

**Figure 2.**
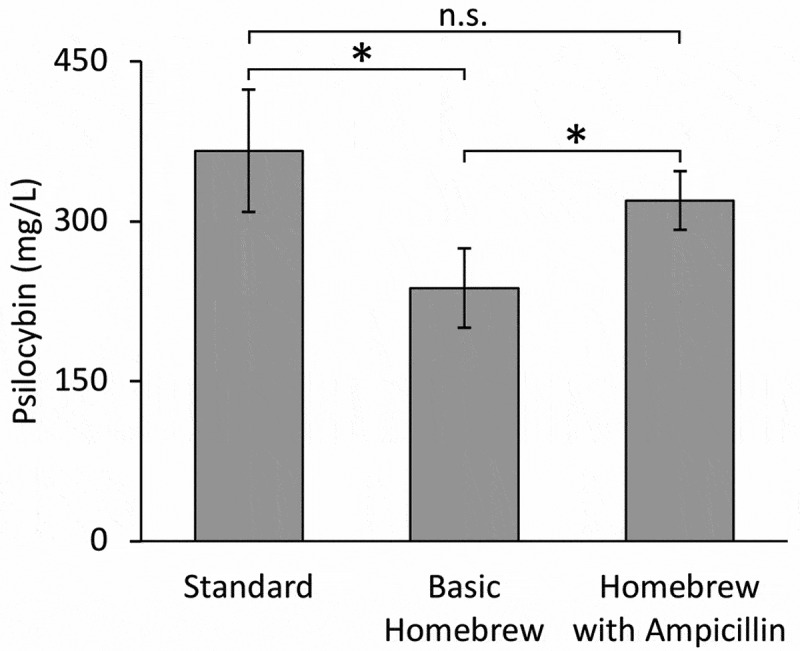
Psilocybin titers under each set of culture conditions. * denotes significant difference, p < 0.01; n.s. denotes no significant difference, p > 0.1

Standard Conditions produced the highest average titer at 366 ± 67 mg/L of psilocybin (28.6% molar yield on 4-hydroxyindole), followed by the Homebrew with Ampicillin Conditions producing an average titer of 319 ± 27 mg/L of psilocybin (28.6% molar yield on 4-hydroxyindole), while the Basic Homebrew Conditions produced the lowest average titer out of the conditions tested at 247 ± 34 mg/L of psilocybin (25.7% molar yield on 4-hydroxyindole) (Figure 2). The addition of ampicillin to the Basic Homebrew Conditions improved titers significantly (p < 0.01), presumedly due to better plasmid stability and less microbial competition under ampicillin selection. Several other trials, with varied media compositions and conditions, produced even higher titers of psilocybin, thus demonstrating that titers could be increased with further optimization (data not shown). These conditions were not pursued due to the higher level of process complexity, which was not appropriate for our simple homebrew environment simulation. Raw data and the results of statistical tests of significance can be found in Supplementary File 1.

A cost analysis was performed to determine the economic feasibility of homebrewed psilocybin. A typical heavy recreational dose of ‘magic’ mushrooms is 1/8^th^ of an ounce and has street value of about $35. This mass of dried mushrooms contains approximately 30 mg of psilocybin [39]. One liter of culture broth fermented under the Homebrew with Ampicillin Conditions contains nearly 11 doses, equal to $385 in dried mushrooms. With the exception of the starting substrate, 4-hydroxyindole, all materials and supplies used were sourced from widely available, unregulated, online vendors. We attempted to assess the ability of individuals to purchase 4-hydroxyindole from chemical companies as described in the discussion. The equipment capital costs were estimated to be roughly $500 USD. While this is more expensive than traditional mushroom cultivation, it is not high enough to be cost prohibitive for the average individual. Furthermore, the operating costs are estimated to be approximately 50x lower than the equivalent street value of psilocybin mushrooms, leading to a short payback period for the capital expenses. It is important to note that this analysis does not consider the costs associated with downstream purification of psilocybin from the cell broth, as analysis of purification techniques is considered beyond the scope of this study. This analysis, coupled with the scalability and speed of fermentation-based production, which far exceeds that of mushroom cultivation, motivates the recommendations of this study presented below.

## 4. Discussion:

Here, we demonstrate the ability to produce biologically relevant quantities of the Schedule I controlled substance, psilocybin, using mostly easy to source items in a mimicked homebrew style environment. This work demonstrates the biosynthesis of psilocybin at concentrations in the 100s of mg/L are possible even when the sterile techniques and equipment common to a research laboratory environment are disregarded. Furthermore, the significant reduction (p < 0.01) in psilocybin production observed under Basic Homebrew conditions was rescued through the single addition of ampicillin resulting in no significant difference between Standard and Homebrew with Ampicillin conditions (p > 0.15). The robustness of this bioprocess is a motivating factor for the recommendations presented herein for the regulation of psilocybin biosynthesis technologies.

**Figure 3.**
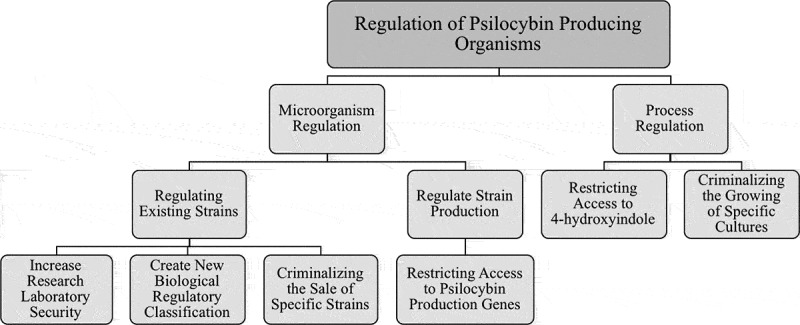
Flow chart illustrating possible regulatory points

Homebrewed psilocybin is possible, provided that one is able to acquire a specific recombinant microbial strain and the required chemicals for the culture media and substrate supplementation. Growing interest in the pharmaceutical efficacy of psilocybin has fueled public interest in ‘magic’ mushrooms, leading to greater curiosity from the general public in this illicit recreational drug. This interest, coupled with recent technological advances for its biosynthesis, warrants a reevaluation of current policies to control access to this powerful chemical. We have identified two main routes to limiting production outside a basic research or clinical laboratory setting from successfully homebrewing psilocybin: microorganism regulation and process regulation (Figure 3).

The most direct path toward limiting psilocybin homebrewing efforts is through microorganism regulation. In the case of *de novo* production, as achieved from *Aspergillus nidulans* [28] and *Saccharomyces cerevisiae* [30], limiting access to the physical microorganism is the only mechanism limiting strain misuse. Acquiring these genetically enhanced organisms could be as simple as an individual obtaining a sample of the recombinant strain from a lab. Enacting appropriate security and diversion control measures at the physical lab facilities is a way to limit organism access. This can be accomplished using multiple layers of locks and badge access restrictions, along with careful inventory control similar to what is required for Schedule I drug diversion control. Although laboratories actively producing controlled substances are required to maintain elevated security measures, track inventory of controlled substances, and are subject to random site security visits, there are currently no such regulations enforced on microorganisms capable of biosynthesis of controlled substances.

Psilocybin-producing recombinant organisms are not the only target to consider in this space. Biosynthesis hosts for scheduled drugs in the opioid [34] and cannabinoid [40] classes have been reported with others still in development [41]. With an ever-growing list of biological organisms that can produce regulated drugs, we recommend creating a new biological classification for these organisms in order to limit free access to these powerful organisms while continuing to support the important scientific advancements resulting from strain development.

A starting point for this regulation would require the U.S. Drug Enforcement Administration (DEA) or international equivalent to determine where these microorganisms fall on the spectrum of controlled chemical-producing organisms. The spores of ‘magic’ mushrooms are legal in most states in the U.S. and in many countries around the world, and they can be sold on the basis that they do not contain psilocybin or psilocin. Similarly, the seeds of both the cannabis plant and the opium poppy (*Papaver somniferum*) are also commonly legal as long as there is no implied intent to produce illicit products. In this sense, organisms with merely the potential to produce psilocybin would be considered legal as they do not inherently contain regulated chemicals. Alternatively, regulation of genetically modified organisms in a similar manner to that of the cannabis or coca plant would render the recombinant organism illegal. Consideration should be given to this legal dichotomy when drafting guidelines that limit clandestine efforts, while simultaneously supporting and enhancing scientific advancements in the field.

Another option for further regulation of psilocybin biosynthesis is to limit the ability for unaffiliated molecular biologists (e.g., biohackers) to assemble recombinant metabolic pathways containing the necessary pathway genes. To date, there have been three recombinant host strains engineered to biosynthesize psilocybin: *A. nidulans* [28], *E. coli* [29], and *S. cerevisiae* [30]. In each of these examples, some combination of the pathway genes from *P. cubensis*, PsiH, PsiD, PsiK, and PsiM were utilized to facilitate the biosynthesis process. The most direct method of sourcing these gene sequences would be to purchase these sequences from one of many custom gene synthesis companies, followed by standard molecular cloning to assemble a functional pathway. One method to counter this approach is to add relevant gene sequences to the screening software used by companies that sell custom DNA synthesis. This could flag orders with high sequence similarity triggering additional customer screening protocols. Despite efforts to regulate access to the genetic material, much regulation on this front will be futile as access to mushroom tissue from which DNA can be extracted, amplified, and cloned will always be available. For this reason, regulation at the DNA level is not deemed practical and resources can be better allocated toward alternative regulatory agendas.

In addition to regulatory controls on the recombinant microorganisms, clandestine efforts can also be minimized through control of key process chemicals specific to the psilocybin biosynthesis process. Although the large majority of the chemicals that make up the culture media are cheap, widely available, and not specific to psilocybin biosynthesis, there is one key starting substrate, 4-hydroxyindole, that is essential to both the bacterial-based biosynthesis [29] and traditional chemical synthesis approaches [23,27]. 4-hydroxyindole is not currently available in the non-regulated online marketplace, making it a good focal point for regulation. Similarly, other pathway intermediates, including: 4-hydroxytryptophan, 4-hydroxytryptamine, norbaeocystin, baeocystin, and norpsilocin, could also be considered as substrates for psilocybin biosynthesis but are all currently limited in commercial availability and prohibitively expensive and thus were not considered in this work.

Upon investigation, very minimal regulation exists for this chemical. The Environmental Protection Agency (EPA) does not list 4-hydroxyindole as an ‘Extremely Hazardous Substance’ in accordance with emergency planning and community right-to-know act (EPCRA) Section 302 and therefore it is not listed on the ‘Reportable Quantities’ list, which requires declaration to state and local authorities. The EPA’s Toxic Release Inventory (EPCRA Section 313) also does not include 4-hydroxyindole [42]. These minimal governing regulations mean that the guidelines imposed by end suppliers are the only regulation on purchasing 4-hydroxyindole. Discussions with several prominent suppliers of 4-hydroxyindole identified the biggest hurdle to purchase 4-hydroxyindole is the need for a business license or business address required by the respective chemical supply companies prior to shipping. Though, purchasing from these companies does not necessarily guarantee that the buyer’s license or address will be reviewed as there is no legal requirement to do so. When contacted, these companies did not disclose the degree of rigor of the investigations into their consumers. The Terms & Conditions page of one predominant supplier states that ‘if evidence is discovered’ that the customer does not hold the proper qualifications, that the supplier has the right to terminate the purchase with ‘no or only partial reimbursement.’

To test whether consumers could ship 4-hydroxyindole to a residential address, we attempted to make purchases from the websites of two 4-hydroxyindole suppliers using a personal credit card, personal e-mail address, and home mailing address. The first supplier did not have 4-hydroxyindole in stock and canceled the order, while the second supplier responded in writing stating that they do not ship to residential addresses and asked for an updated business mailing address. Although the stated policy was enforced in this instance, it is unclear if a business structure as basic as a single member limited liability company (LLC) would qualify as a business.

A regulatory structure similar to the National Precursor Log Exchange (NPLEx), which regulates the over-the-counter sale of pseudoephedrine, could be appropriate to implement in the case of 4-hydroxyindole. Pseudoephedrine, a common ingredient in cold and allergy medication, can be used to make methamphetamine, a Schedule II controlled substance with highly addictive properties. The NPLEx limits amounts that can be purchased daily and monthly by any one individual. Although NPLEx is not without controversy [43], an extension of this regulatory structure to other drug precursors, such as 4-hydroxyindole, would represent a reasonable and common-sense regulation strategy. The current self-imposed regulatory measures are likely not sufficient to prevent misuse of 4-hydroxyindole. Although regulations on 4-hydroxyindole are not the proverbial ‘silver bullet’, their application in concert with microorganism regulation is a start toward mitigating the risk of homebrewed psilocybin.

When making regulatory decisions, it is imperative to consider the impacts the regulations will have on legal yet overlapping industries. For example, in the case of microorganism regulation, it is important to consider the benefits basic scientific research into natural products and applications of recombinant DNA technology have brought to fields ranging from medicine to agriculture [44]. Regulations that slow, or worse, disincentivize progress in these fields should be carefully monitored and implemented. In the case of regulation on 4-hydroxyindole, psilocybin synthesis is not the only end application. 4-hydroxyindole has applications in hair dyes [45], polymer synthesis [46], and a variety of pharmaceutical and medical products with applications in diabetes [47], HIV [48], cancer [49], and antiviral therapies [50]. These additional uses will need to be kept in mind when developing regulatory policies impacting 4-hydroxyindole.

## 5. Conclusion:

In this work, we have successfully shown that reasonable titers of psilocybin could be produced under simple fermentation conditions, thus demonstrating the need to address current security protocols and best practices when working with relevant engineered microorganisms. We have proposed guidance on regulatory methods for control of both the biological and process aspects of psilocybin production. To that point, we have proposed the creation of a new regulatory class focused on recombinant microorganisms with the capacity to produce regulated chemicals, along with increased monitoring for specific precursor chemical markets. These proposed approaches are still not sufficient to completely block illegal biosynthesis efforts but will slow the development of home-based fermentation processes. Finally, we recommend that these guidelines be considered, not unilaterally, but rather in committee with all relevant parties (scientists, regulatory experts, law enforcement, chemical manufacturers, etc.) present and active in the decision-making process.

## Supplementary Material

Supplemental Material

## Highlights:

- Homebrewed psilocybin is possible and economically competitive with mushrooms
- Simple *E. coli* fermentation setup is able to produce >300 mg/L of psilocybin
- Proposed regulation of recombinant microorganisms and key chemical precursors
