# Supplementary material for: Homebrewed psilocybin: can new routes for pharmaceutical psilocybin production enable recreational use?
Source: Bioengineered. 2021 Oct 26;12(1):8863–71. doi: 10.1080/21655979.2021.1987090 (PMC8806991; doi:10.1080/21655979.2021.1987090)
Supplement: Supplemental Material [file KBIE_A_1987090_SM7301.docx]

Supplementary Materials for

**Homebrewed Psilocybin: Can New Routes for Pharmaceutical Psilocybin Production Enable Recreational Use?**

**Authors**

William J. Gibbons Jr.^1,†^ , Madeline G. McKinney^1,†^, Philip J. O’Dell^2,†^, Brooke A. Bollinger^1,†^, J. Andrew Jones^1,*^

**Affiliations**

^1^Miami University, Department of Chemical, Paper, and Biomedical Engineering, Oxford, OH 45056.

^2^Miami University, Department of Chemistry and Biochemistry, Oxford, OH 45056.

^†^These authors contributed equally to this work.

***Corresponding Author Information**

J. Andrew Jones

Miami University

66D Engineering Building

650 E. High St. Oxford, OH 45056

(513) 529-0756

USA

**This PDF file includes:**

Tables S1 to S4

Table S1.

Results from homebrew conditions evaluation studies (N=6).

|  | Standard (mg/L) | Homebrew (mg/L) | Ampicillin (mg/L) |
| --- | --- | --- | --- |
| #1 | 362.8 | 241.3 | 295.2 |
| #2 | 428.0 | 189.7 | 353.5 |
| #3 | 354.8 | 274.0 | 301.4 |
| #4 | 451.0 | 278.4 | 331.9 |
| #5 | 334.0 | 270.4 | 290.8 |
| #6 | 266.0 | 229.7 | 343.5 |
| Average | 366.1 | 247.2 | 319.4 |
| STDEV | 66.7 | 34.3 | 26.9 |

Table S2.

Two-tailed, unpaired t-Test Result comparing Standard and Homebrew conditions.

| t-Test: Two-Sample Assuming Unequal Variances | | |
| --- | --- | --- |
|  |  |  |
|  | *Standard* | *Homebrew* |
| Mean | 366.1 | 247.2 |
| Variance | 4445.8 | 1176.5 |
| Observations | 6 | 6 |
| **P(T<=t) two-tail** | **0.0060** |  |
| t Critical two-tail | 2.3646 |  |

Table S3.

Two-tailed, unpaired t-Test Result comparing Standard and Ampicillin conditions.

| t-Test: Two-Sample Assuming Unequal Variances | | |
| --- | --- | --- |
|  |  |  |
|  | *Standard* | *Ampicillin* |
| Mean | 366.1 | 319.4 |
| Variance | 4445.8 | 725.1 |
| Observations | 6 | 6 |
| **P(T<=t) two-tail** | **0.1554** |  |
| t Critical two-tail | 2.3646 |  |

Table S4.

Two-tailed, unpaired t-Test Result comparing Homebrew and Ampicillin conditions.

| t-Test: Two-Sample Assuming Unequal Variances | | |
| --- | --- | --- |
|  |  |  |
|  | *Homebrew* | *Ampicillin* |
| Mean | 247.2 | 319.4 |
| Variance | 1176.5 | 725.1 |
| Observations | 6 | 6 |
| **P(T<=t) two-tail** | **0.0029** |  |
| t Critical two-tail | 2.2622 |  |
